# Impulsivity assessed ten years earlier and sociodemographic factors predict adherence to COVID-19 related behavioral restrictions in old individuals with hypertension

**DOI:** 10.1186/s12889-022-12624-z

**Published:** 2022-01-27

**Authors:** Patrizia Steca, Roberta Adorni, Andrea Greco, Francesco Zanatta, Francesco Fattirolli, Cristina Franzelli, Cristina Giannattasio, Marco D’Addario

**Affiliations:** 1 Department of Psychology, University of Milano-Bicocca, Piazza dell’Ateneo Nuovo 1, 20126 Milan, Italy; 2 Department of Human and Social Sciences, University of Bergamo, Bergamo, Italy; 3 Department of Medical and Surgical Critical Care, Cardiac Rehabilitation Unit, University of Florence, Florence, Italy; 4 Azienda Ospedaliero-Universitaria Careggi, Florence, Italy; 5 Cardiac/Pulmonary Rehabilitation, ASST Gaetano Pini – CTO, Milan, Italy; 6 School of Medicine and Surgery University of Milano-Bicocca, Milan, Italy; 7 Cardiology IV, “A. De Gasperis” Department, Ospedale Niguarda Ca’ Granda, Milan, Italy

**Keywords:** Health education, COVID-19, Pandemic, Gender, Risk and protection factors, Impulsivity, Type a personality, Adherence to behavioral restrictions

## Abstract

**Background:**

The COVID-19 pandemic has had clear and dramatic repercussions on health, the economy, and psychosocial well-being. Behavioral measures, such as wearing facemasks and maintaining distance from others, have proven crucial in fighting the contagion’s spread. This study aimed to investigate Type A personality traits and sociodemographic predictors of adherence to governmental measures in a sample of frail individuals.

**Methods:**

A sample of 105 Italians over age 60 (Mean age = 70 years; 60.6% male) affected by hypertension who participated in a previous longitudinal study were assessed through a telephone structured interview. Sociodemographic information and Type A personality traits were retrieved from the original longitudinal study. Adherence behaviors were investigated through several questions regarding the compliance with home confinement, the use of facemasks and the observance of social distancing. Repeated measures Analyses of Variance (RMANOVA), Reliable Change Index, and binomial logistic regression analysis were performed.

**Results:**

Only 33.3% of the participants reported adherence to all the governmental COVID-19 measures. Being a woman (OR = 4.84; 95% CI = 1.58, 14.90; *p* < 0.01), being retired (OR = 4.89; 95% CI = 1.09, 21.86; *p* < 0.05), and suffering from hypertension for a relatively short time (OR = 4.20; 95% CI = 1.22, 14.44; *p* < 0.05) positively predicted adherence to the governmental measures. Impulsivity resulted in a stable personality characteristic over the last ten years (*p* = 0.30). Having high levels of impulsivity (OR = 2.28; 95% CI = 1.13, 4.59; *p* < 0.05) negatively predicted adherence.

**Conclusions:**

Our results demonstrate that impulsivity is a stable personality facet that can have a robust negative impact on adherence behaviors to health claims. Overall, results show the importance to tailor communication strategies that consider the role of sociodemographic indicators and impulsivity to achieve a high level of adherence.

## Background

Since the beginning of 2020, the COVID-19 pandemic has had severe repercussions on people’s lives worldwide [1]. A drastic and radical shift, which touched the entire world population whether they caught the virus or not, was the change in one’s daily habits. Indeed, several countries implemented a series of drastic measures to avoid overloading their health systems and delay the spread of contagion [2, 3].

Italy was the first European country that had to face the pandemic. On March 9, 2020, the government enforced lockdown measures on the entire nation [4]. Measures included travel restrictions, the mandatory closure of schools, nonessential commercial activities, and industries. People could only leave their homes for essential reasons, such as going to a medical appointment, a drugstore, or a supermarket. These restrictions were associated with precise behavioral indications (e.g., washing hands frequently, social distancing, using facemasks) widely recognized as the only effective strategy available to counter the spread of the contagion at the time [5, 6].

In such a dramatic situation, the success of government measures depends on the population’s willingness to adhere to them actively [7]. While some people perceive them as a heavy psychological burden resulting in frustration and anxiety, other people try to maintain their daily routine as much as possible and do their best to adapt to the current situation [8].

Adherence to health claims is a significant issue in contemporary medicine as it is widely recognized that individual behavior plays a pivotal role in several communicable and non-communicable diseases [9, 10]. An extensive literature has demonstrated the role of personality in adherence to health claims. Since the 1950s, two American cardiologists, Friedman and Rosenman, explored the relationship between some personality characteristics and the incidence of cardiovascular diseases, coming to identify the so-called Type A personality typology [11, 12]. Individuals with marked Type A characteristics exhibit impatience, impulsivity, a sense of time urgency, competitiveness, striving for achievement, aggressiveness, and restlessness [11]. In the 70s and 80s Type A personality typology was at the center of studies on the link between personality and cardiovascular diseases [13, 14]. Over the years, several studies have confirmed an association between the Type A personality and worse health conditions, particularly cardiovascular diseases and diabetes [15–19]. However, many other studies have obtained contrasting results [20, 21].

Interestingly, in a recent systematic review, Mommersteeg and Pouwer [22] evidenced that Type A personality may lead to an increased risk of cardiovascular diseases and diabetes via the mediating role of unhealthy behaviors. These behaviors may represent one of the elements of a causal chain that link personality and poor health conditions. In the same vein, in a previous study on patients with cardiovascular diseases, it was found that patients with Type A profiles adhered less to medical indications to foster a healthy lifestyle, being more physically inactive, and smoking more than patients with a different profile [23].

Among the Type A personality dimensions, impulsivity plays a critical role in adherence behaviors [24, 25]. Impulsivity is a personality facet reflecting a deficit in inhibitory control associated with increased sensitivity to an immediate reward [26]. Opting to adhere or not can be seen as a choice between a larger delayed reward for adherence (e.g., prevention of a disease) and a smaller but more immediate reward for non-adherence (e.g., an extra portion of a favorite food, a cigarette, or in the current pandemic situation, going out for a walk and meeting with others) [27]. Interestingly, emerging evidence suggests that impulsivity is associated with non-adherence with COVID-19-related public health measures. Indeed, a recent cross-sectional study explored to what extent a sample representative of the general American population continued to adhere to social distancing measures in the period after the first lockdown [7]. The authors evidenced that higher impulsivity was associated with lowering adherence to social distancing measures. In a similar cross-sectional study focused on the general Dutch population [28], the authors found that people with higher impulsivity were more likely to violate the rules. Again, a recent longitudinal study focused on a cohort of young Swiss adults [29] evidenced that non-adherence with COVID-19 behavioral restrictions was higher in those who had previously scored high on indicators of low self-control, including impulsivity.

The current study aims to contribute to this very recent literature, investigating the role of impulsivity as a longitudinal predictor of adherence. We explored the psychological and sociodemographic predictors of adherence to the Italian government’s behavioral restrictions during the first lockdown in a cohort of 105 patients over 60 with hypertension who had taken part in a previous longitudinal study.

We focused on individuals over 60 with hypertension because older individuals with underlying chronic conditions are more likely to develop severe forms of COVID-19 [30]. These individuals should be the first to follow the government’s restrictions to delay the spread of COVID-19 and avoid overloading the health system. Therefore, it is essential to clarify whether they adhered sufficiently to restrictions and identify the sociodemographic and psychological predictors of their behaviors to plan effective communication strategies for this “frail” segment of the population.

We used a structured interview to evaluate a series of adherence behaviors in depth and reach an older population segment, which is unfamiliar with the internet and therefore difficult to reach through online questionnaires widely used in previous pandemic-focused studies [7, 8, 28, 29, 31–34]. Therefore, the focus on this population segment offered the opportunity to verify whether the psychological and demographic variables that have proven relevant in the general population also play a role in the elderly population with a chronic disease.

Data from the previous study offered the unique opportunity to assess whether personality characteristics, assessed ten years before the pandemic onset, could play a role in determining adherence.

First, we aimed to test the preliminary hypothesis that Type A characteristics will not change over time, according to prior literature [35]. Second, based on a previous study demonstrating that people with higher Type A characteristics reported lower levels of health-promoting behaviors [23], we hypothesized lower adherence to restrictions in these individuals. Specifically, based on the emerging literature [7, 28, 29], we hypothesized adherence would be lower among more impulsive individuals. Third, based on prior literature, we looked at gender [8, 29, 31–33] and occupation [7, 33, 34] as relevant variables shaping how people respond to behavioral restrictions. We hypothesized adherence to be lower among men and working people.

## Methods

### Participants and procedure

The present study involved 105 participants of a previous extensive longitudinal study aimed at profiling patients with essential arterial hypertension in terms of a series of behavioral, clinical, and psychological variables [36].

Regarding the original longitudinal study, patients were firstly recruited between February 2011 and May 2014, based on their access to a large hospital in Northern Italy. Eligible patients were between 30 and 75 years of age, had essential arterial hypertension (i.e., they were already receiving pharmacological treatment or had elevated blood pressure values, including systolic blood pressure (SBP) > = 140 mmHg or diastolic blood pressure (DBP) > = 90 mmHg) and had sufficient Italian language skills. Patients with cognitive deficits or other major pathologies (such as cancer) were excluded. Some participants were involved in the study cross-sectionally. The remaining participants were involved in the study longitudinally and were assessed at four time-points (baseline, 6-, 24-, and 36-months). Data were collected using self-report questionnaires administered to the participants by a trained researcher.

**Fig. 1:**
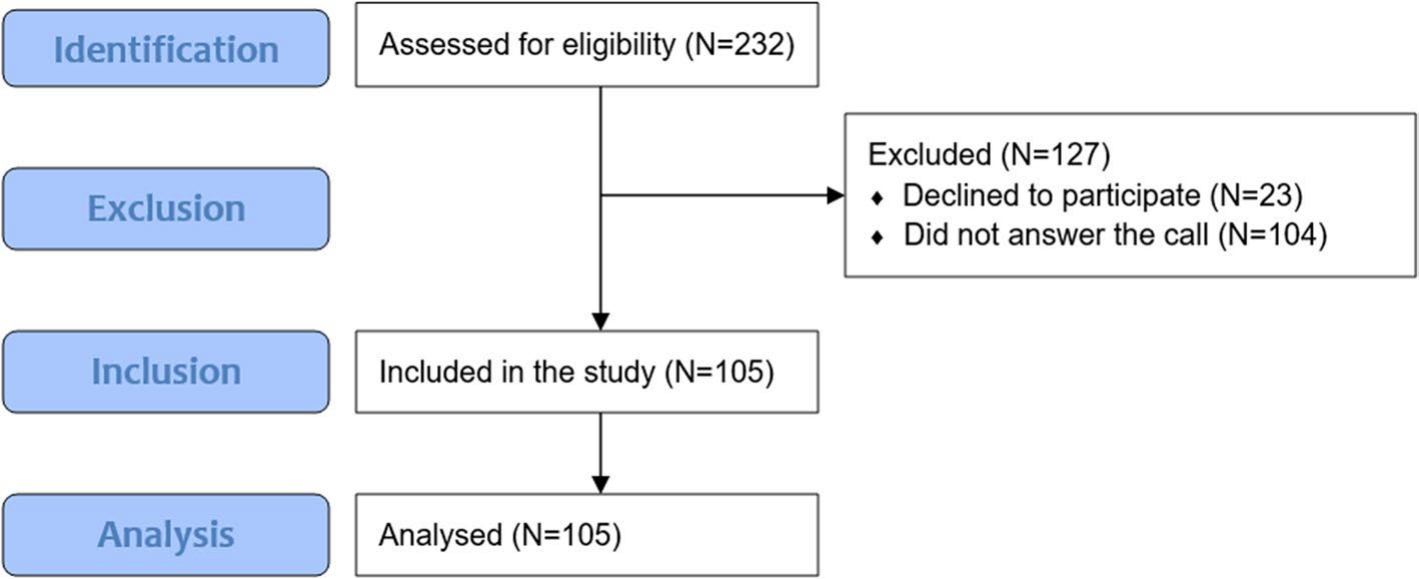
STrengthening the Reporting of OBservational studies in Epidemiology (STROBE) flow chart of study participants

For the present study, we extracted a subsample of eligible patients (both from the cross-sectional and longitudinal samples) from the original study database. In addition to the inclusion criteria already mentioned regarding the longitudinal study, we decided to include only patients over 60. In addition to their clinical condition, the patients’ age range made it possible to identify the sample as constituted by a frail segment of the population, referring to the possible severity of a COVID-19 infection [30]. Of the 232 individuals contacted by telephone, 127 did not participate in the study: 104 did not answer the call and could not be reached, or they had passed away, while 23 declined to participate in the study. The remaining 105 patients participated in the study (see Fig. 1). The 23 patients who declined to participate did not differ significantly from the respondents in gender or education but differed in age, being older on average (77 years; SD = 6.66) than participating patients (70 years; SD = 5.83; *p* < 0.001). The non-participating patients did not differ significantly from the respondents in the Type A personality dimensions (impulsivity, competitiveness, hostility, leadership, and job involvement), except for hostility. The non-participating patients had a higher mean score of hostility (mean = 3.83; SD = 0.53; *p* < 0.05) than participating patients (mean = 3.34; SD = 0.85).

The 105 participants had a mean age of 70 years (SD = 5.83) and were primarily male (60.6%). The proportion of men in the sample was a direct consequence of cardiovascular disease incidence, which is more common among men than women [37]. Most participants were retired (73.1%), had a high school diploma (53.8%), and lived with others (87.5%). Most participants had suffered from hypertension for more than ten years (76.0%).

All participants were given a short telephone interview to detect their condition concerning COVID-19 and their adherence to behavioral indications and national restrictions to contain the pandemic. The phone calls were made using contact information acquired in the previous longitudinal study by staff trained in Psychology with clinical population research experience. Telephone interviews were conducted from the end of May to the beginning of August 2020, immediately after the first lockdown in Italy [4]. According to epidemiological data confirmed by the World Health Organization [1], the investigated timeframe corresponds to the first contagion peak in Italy. Sociodemographic and Type A personality indicators were taken from the longitudinal study dataset.

The Ethical Committee of the authors’ university approved both the original longitudinal study and the present study. All participants received written information about the study and signed a consent form to participate.

The sample size’s adequacy was established by resorting to Power Analysis [38] using the software G*Power Version 3.1.9.7 [39]. We calculated the sample size requested to perform logistic regression with the following parameters: Odds ratio = 3.47, α = 0.05, Power = 0.8. The sample size calculated was 44 individuals. Based on these considerations, the sample size of the study was sufficient to detect small-medium size effects.

### Demographic and clinical indicators

At the beginning of the telephone interview, the participants were asked to report their updated sociodemographic information, particularly living status (alone vs. with others) and employment status (working vs. not working). The interviewer knew a priori the gender, age, and educational level of the participants. The participants were also asked to report their general health status and whether they had developed cardiovascular or other diseases since the last assessment.

### Adherence to behavioral indications and national restrictions

Regarding COVID-19, the participants were asked to report whether they had ever tested positive for the virus and, if so, the severity of their condition (whether they had been hospitalized, whether they had been in intensive care, and the duration of their illness). The same question was asked about their loved ones. Participants provided yes / no answers for each question posed by the interviewer.

The telephone interview continued with a series of questions aimed at investigating adherence to the indications about local and national restrictions during the first lockdown phase (March 9 to May 4) and the subsequent phase (starting from May 4). The questions explored whether the participants had left their houses, for what reasons (i.e., shopping, work, other), and with what frequency. They were also asked if they had reduced their social contacts (particularly if they had met people outside their household), if they kept at least one meter away from other people when they met them (for example, when shopping or working), and if they wore facemasks. They were then asked how often they washed their hands and cleaned objects, clothes, and their home and how much effort they put into implementing all the behaviors required by the government. The Appendix reports the list of questions asked during the telephone interview and a summary of the participants’ responses.

For each of the behaviors investigated in the interview, a dichotomous variable was created that identified adherence (score of 0) or non-adherence (score of 1) to the indications provided by the government. We created a final dichotomous variable that synthesized adherence behaviors. Considering the relevance of maximally adhering to behavioral restrictions, we compared those who fully adhered with those who did not. Participants who had complied with all the Italian government’s restrictions and behaviors were identified as adherents (score of 0). Participants who did not comply with at least one of the government’s restrictions or behaviors were identified as non-adherents (score of 1).

### Type a personality typology

As already mentioned, for Type A personality, we used data previously collected at baseline and three following time-points of a longitudinal study. We used 12 items from the Cognitive Behavioural Assessment Form Hospital battery (CBA-H) [40, 41] and two new researcher-constructed items [23] to evaluate the following five subdimensions: leadership (3 items, for example, ‘When I am with others, I like to be considered a boss or a leader’), competitiveness (2 items, for example, ‘Nothing is achieved in life without being competitive’), hostility (3 items, for example, ‘I am often suspicious of the intentions of others’), job involvement (2 items, for example, ‘I am - or was if retired or unemployed - very involved in employment matters’), and impulsivity (2 items, for example, ‘I get impatient with people who do not understand things quickly’). New researcher-constructed items were ‘I am often suspicious of the intentions of others’ (hostility) and ‘I am convinced that most people only think about themselves’ (hostility). Each item was rated on a 5-point scale, ranging from 1 (Absolutely false for me) to 5 (Absolutely true for me). Each dimension’s score was calculated as the mean item score, where a higher score indicates a higher Type A personality. Validity and reliability statistics for this scale were previously demonstrated and are reported in the study by Steca et al. [23].

### Statistical analyses

To preliminarily evaluate Type A personality changes over time, five repeated measures Analyses of Variance (RMANOVA) were performed. Each analysis considered the mean score of a Type A personality dimension as the dependent variable and time as the independent variable (4 levels: baseline, 6-, 24-, and 36-months).

To support the results of RMANOVAs, we also examined whether mean-level continuity extended to the individual level, examining the number of individuals showing decreased, equal, or increased trait scores. We used the RCI (Reliable Change Index), an index developed to assess the clinical significance of change after therapeutic intervention [42]; this index is also used to determine how many individuals remain stable on their personality pattern across time [43]. For the calculation of the RCI, we considered, for each personality dimension, the difference between the average score assessed at the baseline and at the most distant time-point available (36-months). Then, we divided the resulting figure by the standard error of the difference between the test scores. We considered the patient change reliable when it exceeded the measurement error at a 0.05 level of confidence.

A binomial logistic regression analysis was performed, with the summary variable “adherence behaviors” as the dependent variable (2 levels: adherent, non-adherent) and the five dimensions of Type-A typology as covariate predictors. Demographic variables, namely gender, age, educational level (less than high school vs. high school or higher), living status (alone vs. with others), employment status (working vs. not working), and time of illness (participants who had suffered from hypertension for more than ten years - before enrollment in the longitudinal study - vs. less than ten years) were included as categorical (gender, educational level, living status, employment status, and time of illness) or covariate (age) predictors in the regression analysis to consider their potential effect on adherence to the indications provided by the government. A *p* value ≤.05 was considered statistically significant.

The analyses were performed using the Statistical Package for the Social Sciences (SPSS) software, version 26.

## Results

### Participants’ health condition

Overall, 92% of the participants (97 patients out of a total of 105) reported good general health, even though 23 had undergone some kind of treatment that required hospitalization in the period between the last assessment of the longitudinal study and the telephone interview. Fourteen patients had cardiovascular events; six had oncological events, and three had pneumatological events. None of the participants had ever been diagnosed with COVID-19 at the survey time. Most of them (83.7%) said they had never contracted the virus, while the remaining 16.3% said they did not know if they had contracted the virus. Most of the participants (75.5%) said they did not know someone who had been diagnosed with COVID-19; the remaining 24.5% declared that they knew someone who had been diagnosed with COVID-19.

### Type a personality stability

The analysis performed considering the mean score of each Type A personality dimension as the dependent variable and time as the independent variable showed no significant effect of time, suggesting that the five dimensions did not change over time (Table 1).

**Table 1:** Results of the repeated measures ANOVAs analyzing possible Type A personality changes over time (Italian patients over 60 with hypertension; 2020)

	N	Baseline Mean (SD)	6-months Mean (SD)	24-months Mean (SD)	36-months Mean (SD)	F	Sign.
Leadership	95	2.48 (0.75)	2.56 (0.73)	2.47 (0.74)	2.54 (0.70)	0.88	0.45
Competitiveness	95	2.90 (0.98)	2.92 (0.97)	3.02 (0.82)	2.93 (0.87)	0.77	0.50
Hostility	95	3.35 (0.87)	3.37 (0.75)	3.36 (0.72)	3.37 (0.74)	0.04	0.99
Job involvement	90	3.65 (0.97)	3.54 (0.86)	3.41 (0.80)	3.48 (0.88)	2.58	0.06
Impulsivity	95	2.7 (0.90)	2.8 (0.83)	2.73 (0.77)	2.65 (0.79)	1.23	0.30

The results of the individual-level continuity of type A personality confirmed that no change occurred over time in most patients (Table 2).

**Table 2:** Results of the individual-level continuity of type A personality based on RCI (Italian patients over 60 with hypertension; 2020)

	No change	Increase	Decrease	No sufficient data available^a^
**Leadership** ***(n, %)***	92 (88%)	–	–	13 (12%)
**Competitiveness** ***(n, %)***	88 (84%)	2 (2%)	2 (2%)	13 (12%)
**Hostility** ***(n, %)***	88 (84%)	1 (1%)	3 (3%)	13 (12%)
**Job involvement** ***(n, %)***	87 (83%)	0 (0%)	3 (3%)	15 (14%)
**Impulsivity** ***(n, %)***	91 (87%)	0 (0%)	1 (1%)	13 (12%)

*Note*. ^a^Participants in this category were those recruited for cross-sectional assessment in the original study. Therefore, no longitudinal personality data were available

The results of these two analyses allow us to conclude that in the present study sample, the dimensions of the type A personality can be considered stable personality characteristics. Therefore, we considered the mean item scores of the dimensions measured at baseline in the following analyses.

### Adherence to prescriptions

**Fig. 2:**
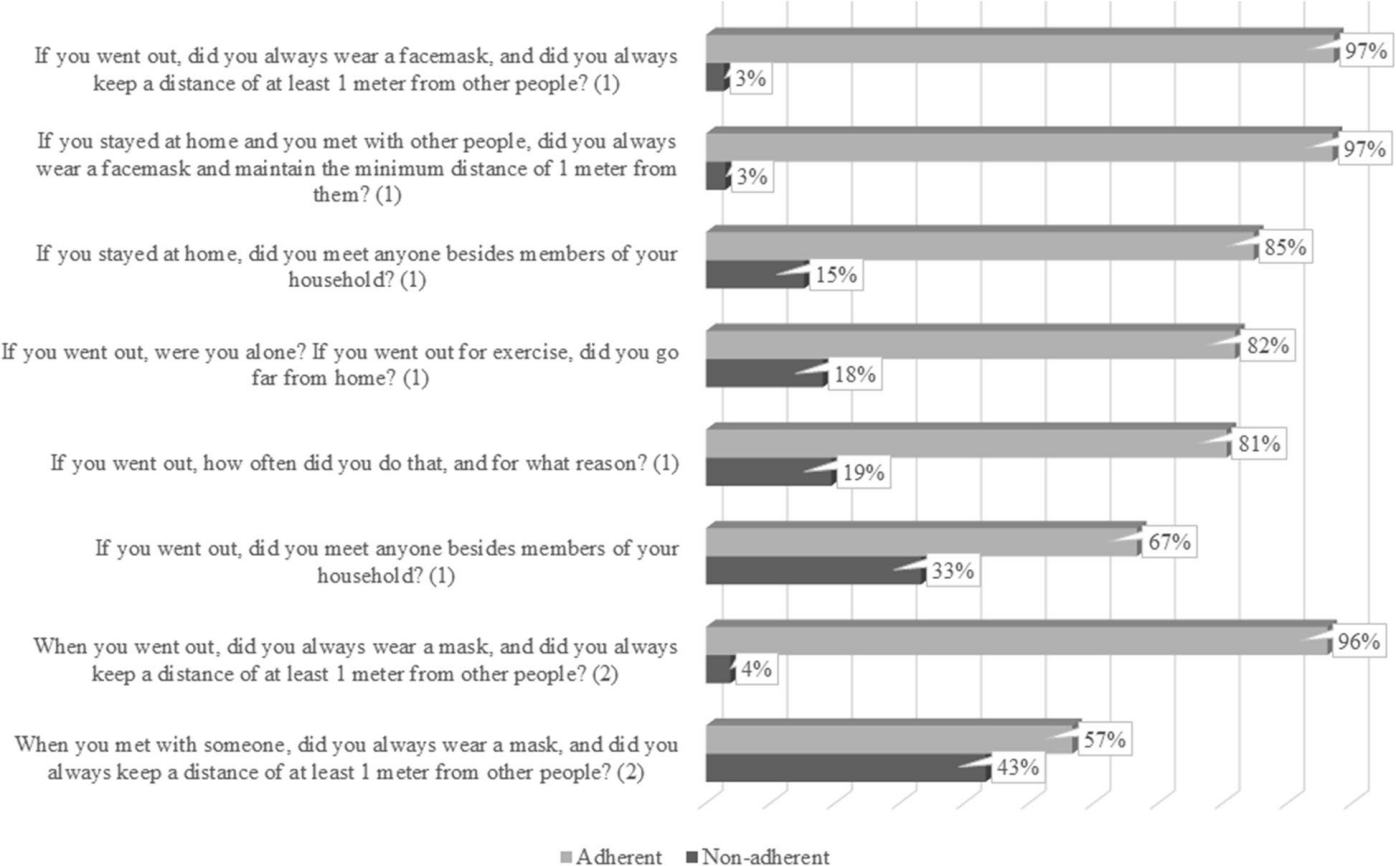
Percentage of participants who adhered/did not adhere to the Italian government’s restrictions (*N* = 105; Italian patients over 60 with hypertension; 2020). *Note*. (1) Phase 1 (March 9th-May 4th); (2) Phase 2 (after May 4)

A third of the participants (33.3%) declared that they were complying with all the restrictions and behaviors imposed by the Italian government (adherents); the remaining 66.7% declared that they were not complying with at least one of the restrictions or behaviors imposed by the government (non-adherents). Figure 2 illustrates the list of questions asked during the telephone interview and the percentage of participants who adhered/did not adhere to the government’s COVID-19 indications and restrictions during the two phases of the lock-down in Italy.

### Prediction of adherence to restrictions

The binomial logistic regression results indicated that the full model containing all predictors was statistically significant, χ^2^(11, *N* = 95) = 28.4, *p* < .005, indicating that the model was able to distinguish between respondents who were adherent to the behavioral restrictions vs. those who were not. The model explained between 25.9% (Cox & Snell’s R^2^) and 35.7% (Nagelkerke’s R^2^) of the variance in adherence to restrictions and correctly classified 80% of the cases. As shown in Table 3, only four independent variables made a unique, statistically significant contribution to the model (gender, occupation, time of illness, and impulsivity). The stronger predictors of reporting non-adherence were gender (*p* < .01) and working status (*p* < .05). The odds ratio (4.84 and 4.89, respectively) indicated that men and working people were five times more likely to report non-adherence than women and non-working people. Results also showed a significant role of time of illness (*p* < .05), recording an odds ratio of 4.20. This indicated that participants who had suffered from hypertension for a longer time were four times more likely to report non-adherence than participants who had suffered from hypertension more recently. Finally, results showed that participants with higher impulsivity scores were less adherent than those with lower impulsivity scores (*p* < .05). The odds ratio (2.28) highlighted that participants were two times more likely to report non-adherence to restrictions for every unit increase in impulsivity.

**Table 3:** Binomial logistic regression analyzing influences of the demographic variables and Type A personality on adherence (N = 95; Italian patients over 60 with hypertension; 2020)

	B	SE.	Wald	df	Sign.	Odds Ratio	95% CI for Odds Ratio	
Age	−0.05	0.05	0.80	1	0.37	0.96	0.87	1.06
Gender	1.58	0.57	7.57	1	0.01	4.84	1.58	14.90
Education	−0.47	0.70	0.45	1	0.50	0.63	0.16	2.46
Currently live with others	1.33	0.93	2.03	1	0.15	3.77	0.61	23.35
Occupation	1.59	0.76	4.32	1	0.04	4.89	1.09	21.86
Time of illness	1.43	0.63	5.17	1	0.02	4.20	1.22	14.44
Leadership	−0.76	0.43	3.20	1	0.07	0.47	0.20	1.08
Competitiveness	0.24	0.36	0.43	1	0.51	1.27	0.63	2.55
Hostility	−0.41	0.42	0.99	1	0.32	0.66	0.29	1.49
Job involvement	0.26	0.33	0.59	1	0.44	1.29	0.67	2.47
Impulsivity	0.82	0.36	5.28	1	0.02	2.28	1.13	4.59

## Discussion

The present study explored the Type A personality and sociodemographic predictors of adherence to government restriction measures during the first lockdown period in Italy in a frail sample of individuals over 60 with hypertension. Using a structured interview, rather than an online questionnaire, and focusing on a clinical population that had taken part in a previous longitudinal study, rather than the general population, offered several strengths and made the study results unique in the panorama of publications on COVID-19 pandemic.

The results show that only one-third of the sample adhered to all the restrictions. This low percentage underlines how difficult it is to adhere to all the containment measures due to their significant impact on people’s daily lives. A similar result has been recently reported in a cross-sectional study focused on a convenience sample of North London’s residents [34]. Only 7.2% of the participants reported being able to adhere to all social distancing rules. The odds of not adhering to all social distancing rules decreased if a participant was identified as highly vulnerable to COVID-19. Nevertheless, our results show that difficulty to adhere to all the containment measures is also true for the elderly and frail population, namely the people who are at higher risk for the virus contraction’s worst consequences.

Regarding the personality predictors of adherence, the results showed that participants with higher impulsivity scores were less adherent than participants with lower scores. Consistently, the role of impulsivity has emerged in studies focused on adherence to medical claims in different domains [25]. Indeed, people with high impulsivity are more likely to overeat [44], or smoke [45], or again, not adhere to prophylaxis over time [24]. Similar results were recently described in two cross-sectional studies that explored to what extent samples representative of the general Dutch [28] or American [7] population adhered to social distancing measures during the first peak of the pandemic [28] and the period after the first lockdown [7]. The authors showed that higher impulsivity levels were associated with lower adherence. Similarly, our study results illustrated that the most challenging behavior to assume was social distancing in both phases of the lockdown. As already mentioned in the introduction, impulsivity is a personality facet reflecting a deficit in inhibitory control associated with increased sensitivity to an immediate reward [26]. More impulsive individuals may experience greater difficulty opting for a larger delayed reward - preventing their own and loved ones from getting infected – and they may focus on a more immediate reward - going out for a walk and meeting with friends or relatives [27]. Our findings add to previous cross-sectional studies focused on adherence with COVID-19-related public health measures showing that impulsivity may be a stable personality characteristic in adulthood [35] and may influence a contingent behavior several years after its assessment. In this regard, it is interesting to underline that our results are also consistent with those of a recent longitudinal study focused on a cohort of young adults of age 22 [29]. The authors found that adherence with COVID-19 related public health measures was lower in young adults who had exhibited high levels of impulsivity in previous years - between the ages of 15 and 20. Impulsivity has been widely investigated in children and adults in different domains of health-related behaviors, but it has received little attention in older adults [46]. Our findings highlight that impulsivity is a personality facet that can play a crucial role in health-related behaviors even in old age.

Regarding the role of sociodemographic indicators, the results showed that men were less adherent than women. This result is in line with recent studies showing that men were at increased risk of refusing to adhere to government measures to contain COVID-19 spread and suggesting that men are generally more likely to engage in risk-taking behaviors [8, 29, 31–33]. It is also in line with previous studies focused on adherence to healthy lifestyle behaviors in a clinical population [47].

The results also showed that working people were less adherent than non-working people. People who continued to work during the lockdown period and therefore had more opportunities to leave their homes may have been more tempted to violate government restrictions. This result is in line with a recent study mentioned above [7]. The authors found that situational variables played a central role in participants adhering to social distancing measures. Recalling the sociological theory of routine activities [48], the authors pointed out that being exposed to the opportunity to break the rules might give incentives to do so [7]. This evidence was also found in two other European cross-sectional studies mentioned above [28, 34] and a Brazilian cross-sectional study [33].

Finally, the results showed that participants who had suffered from hypertension for the longest time were less adherent. Most of the patients interviewed reported good general health; only a small percentage had clinically significant events in recent years. Having had a chronic disease for a long time without experiencing acute and severe events might lead these patients to perceive their health condition as less severe [49] and take recommendations less seriously. This observation is in line with the shared evidence that non-adherence is especially common in ‘silent’ diseases [36, 50].

While this study provided new insights into how personality traits and sociodemographic variables may influence adherence to government restrictions, it has limitations. First, the sample was mostly made up of men over 60 suffering from a specific chronic, non-communicable disease. On the one hand, this limits the generalizability of the results. On the other hand, it allows us to make valid, reliable inferences for this population segment. Notably, the results of the present study enforce the existing evidence that similar links between psychological and sociodemographic variables and adherence to government restrictions can be found across different population segments, countries, and study designs. Future studies could explore the sociodemographic and psychological predictors of adherence in patients with other chronic diseases relevant to the risk of severe complications following COVID-19 infection, such as respiratory diseases.

A second limitation is that the sample size is small; therefore, the statistical power of the analysis is limited. This aspect contributes, together with the previous one, to limit the generalizability of the results.

A final limitation of the present study is that it relies merely on a self-report method. Although methodological and inferential limitations constrain these kinds of studies (e.g., social desirability bias), they are suitable to provide important steps in understanding a phenomenon [51], and they have significant advantages (e.g., high practicality of use, clinical and research applicability, and good cost-effectiveness). Accordingly, recent evidence showed that self-reported and observations of actual behavior during the COVID-19 pandemic overlap [52].

This study also has several strengths. The most important is that personality characteristics were assessed long before the pandemic, and they turned out to be stable over time. Thus, they were not affected by the particular living conditions and psychological burden associated with the pandemic. Our findings showed how much a stable personality characteristic could influence contingent behavior observed years later, and they add a unique contribution to the knowledge on the long-term impact of impulsivity on health-related behaviors during adulthood. Second, the assessment timeframe was immediately after the first pandemic peak. This has offered the possibility to timely collect accurate information on the phenomenon under consideration. Furthermore, unlike most pandemic-focused studies - carried out through online questionnaires - this study employed a telephone interview. This made it possible to focus on a well-defined sample, to evaluate a series of adherence behaviors in depth, and reach a segment of the population that, due to old age and unfamiliarity with the internet, is difficult to reach through questionnaires published online. The final strength of this study is the focus on older individuals with underlying chronic conditions. Considering that these individuals are more likely to develop severe forms of COVID-19 [30], they should be the first to follow the government’s restrictions to delay the spread of COVID-19 and avoid overloading the health system. This study contributes to clarify whether they adhered sufficiently to restrictions and identify the sociodemographic and psychological predictors of their behaviors.

The present study highlighted informative findings for practical implications and future research. Adhering to health recommendations represents not only a protective factor for the individual, especially for the one at higher risk, but it also outlines a central theme on the subject of public health. The detailed observations made in the present study concerning the adherence behaviors during the earliest phases of the pandemic suggest that following health-related indications and restrictions is not always easy. These findings, particularly if we consider the sample involved, warn that more specific and oriented strategies are increasingly needed. For this purpose, health communication may represent a reliable tool for promising solutions to respond effectively to public health issues. To date, extensive research has reported that effective communication on health-related themes is essential for optimal adherence to recommended health behaviors [53]. In particular, it has been shown that the more the information provided is tailored to the personal features, the more effective the communication is in influencing the target behavior [54, 55]. Following this line, our results provided insight into the prediction and impact of specific socio-demographic and psychological factors. Accordingly, future public health interventions should pay more attention to those specific segments of the older population (i.e., men, workers, those suffering from hypertension for long, and those displaying high levels of impulsivity) that were less likely to comply with the governmental restrictions. Health communication campaigns may consider such variables to better tailor the information to transmit to improve individuals’ knowledge and increase the prevalence of positive behaviors. So, the implementation of improved health promotion and communication policies, more sensible to the personal characteristics of the target, may represent a valid solution and consequently benefit the community, especially in the way of COVID-19 prevention. One strategy might be storytelling to stimulate people to change their attitudes toward public health issues [56]. Valuable insights may also come from studies showing that emphasizing the immediate, concrete advantages of being adherent can effectively improve exercise adherence in sedentary adults [57] and reinforce healthy choices in patients prone to impulsivity [58]. Future solutions may be searched in mhealth strategies that implement innovative technological tools (e.g., smartphone apps) that can efficiently deliver tailored content. Such strategies may play a crucial role in obtaining adherent behaviors and, ultimately, in benefiting the community.

## Conclusions

In summary, this study explored Type A personality and sociodemographic predictors of adherence to government restriction measures during the first lockdown in Italy in a sample of individuals over 60 affected by hypertension. Results showed that impulsivity, evaluated as a stable personality characteristic over the years, can predict adherence. They also highlighted how men, working people, and those with chronic silent diseases are more at risk of non-adherence. Therefore, results show the importance of adopting effective communication strategies tailored to specific population segments from whom a high level of adherence is more challenging to obtain.

## Data Availability

The datasets used and/or analyzed during the current study are available from the corresponding author on reasonable request.
